# 25,26-Bis(propan-2-yl­idene)hepta­cyclo[20.2.1.1^10,13^.0^2,21^.0^3,8^.0^9,14^.0^15,20^]hexa­cosa-2(21),3,5,7,9(14),11,15,17,19,23-deca­ene

**DOI:** 10.1107/S1600536812018211

**Published:** 2012-04-28

**Authors:** Stefan M. Cooper, Tamara R. Schaller Nauman, Frank R. Fronczek, Steven F. Watkins

**Affiliations:** a Department of Chemistry, Louisiana State University, Baton Rouge LA 70803-1804, USA

## Abstract

In the title compound, C_32_H_28_, the central cyclo­octa­tetra­ene ring has a boat conformation, and the mol­ecule is saddle shaped. The seat is defined by the mean plane of the four-atom attachment points (r.m.s. deviation = 0.014 Å) of the two bicyclo­heptenyl substituents. These substituents comprise the pommel and cantle, with each mean plane defined by four atoms proximate to the seat (r.m.s. deviations = 0.002 and 0.004 Å). Relative to the seat, the pommel and cantle bend up 31.16 (4) and 29.40 (5)°, while the benzo units (flaps, r.m.s. deviations = 0.006 and 0.009 Å) bend down 36.75 (4) and 38.46 (4)°. The mean planes of the dimethyl­ethyl­idene units are almost perpendicular to the saddle seat, making dihedral angles 86.89 (4) and 88.01 (4)°.

## Related literature

For related structures, see: Durr *et al.* (1983 ▶); Sygula *et al.* (2007 ▶). For the synthesis, see: Schaller (1994 ▶).
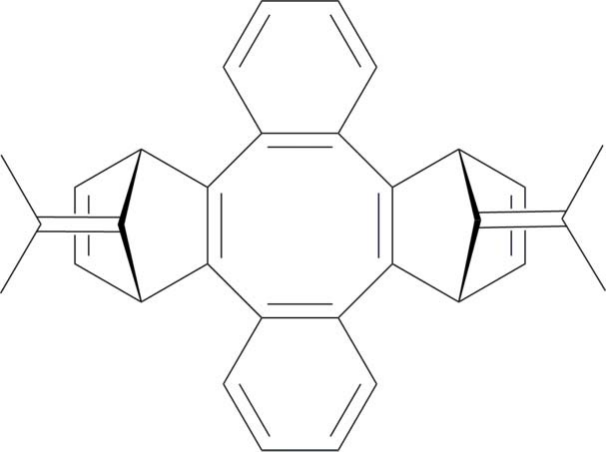


## Experimental

### 

#### Crystal data

C_32_H_28_
*M* *_r_* = 412.54
Triclinic, 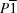

*a* = 9.3577 (2) Å
*b* = 9.5500 (3) Å
*c* = 12.6946 (3) Å
α = 94.068 (2)°
β = 94.402 (2)°
γ = 100.162 (1)°
*V* = 1109.24 (5) Å^3^
*Z* = 2
Mo *K*α radiation
μ = 0.07 mm^−1^
*T* = 90 K
0.40 × 0.30 × 0.27 mm

#### Data collection

Nonius KappaCCD diffractometer
Absorption correction: multi-scan (*SCALEPACK*; Otwinowski & Minor, 1997 ▶) *T*_min_ = 0.973, *T*_max_ = 0.982
15041 measured reflections
7973 independent reflections
5906 reflections with *I* > 2σ(*I*)
*R*_int_ = 0.033

#### Refinement

*R*[*F* ^2^ > 2σ(*F* ^2^)] = 0.052
*wR*(*F* ^2^) = 0.140
*S* = 1.04
7972 reflections
293 parameters
H-atom parameters constrained
Δρ_max_ = 0.40 e Å^−3^
Δρ_min_ = −0.31 e Å^−3^

### 

Data collection: *COLLECT* (Nonius, 2000 ▶); cell refinement: *SCALEPACK* (Otwinowski & Minor, 1997 ▶); data reduction: *DENZO* (Otwinowski & Minor, 1997 ▶) and *SCALEPACK*; program(s) used to solve structure: *SHELXS86* (Sheldrick, 2008 ▶); program(s) used to refine structure: *SHELXL97* (Sheldrick, 2008 ▶); molecular graphics: *ORTEP-3 for Windows* (Farrugia, 1997 ▶); software used to prepare material for publication: *WinGX* (Farrugia, 1999 ▶).

## Supplementary Material

Crystal structure: contains datablock(s) global, I. DOI: 10.1107/S1600536812018211/tk5087sup1.cif

Structure factors: contains datablock(s) I. DOI: 10.1107/S1600536812018211/tk5087Isup2.hkl

Supplementary material file. DOI: 10.1107/S1600536812018211/tk5087Isup3.cml

Additional supplementary materials:  crystallographic information; 3D view; checkCIF report

## Figures and Tables

**Fig. 1.**
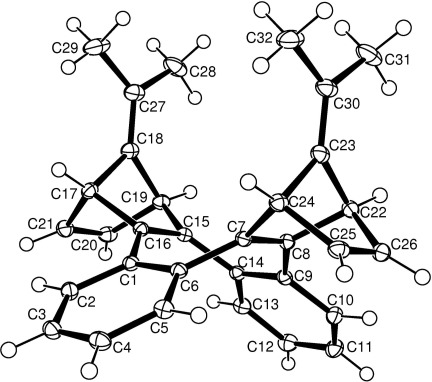
View of (I) (50% probability displacement ellipsoids)

## supplementary crystallographic information

### Comment

Structures related to the title compound have been reported previously (Durr *et al.*, 1983; Sygula *et al.*, 2007). The central 8-ring of the title compound adopts the boat configuration, and the overall shape of the molecule is that of a saddle. Relative to the mean plane of the saddle seat (C7, C8, C15, C16, δ_r.m.s._ = 0.014 Å), the two bicycloheptane moieties (mean planes C7, C8, C22, C24, δ_r.m.s._ = 0.002 Å, and C15, C16, C17, C19, δ_r.m.s._ = 0.0040 Å) bend up 29.40 (5) and 31.16 (4)°, while the mean planes of the benzo moieties (δ_r.m.s._ = 0.010 and 0.012 Å) bend down 36.75 (4) and 38.46 (4)°. The dihedral angles between the saddle seat and the mean planes of the dimethylethylidene moieties (C17, C18, C19, C27, C28, C29, δ_r.m.s._ = 0.006 Å; C22, C23, C24, C30, C31, C32, δ_r.m.s._ = 0.009 Å) are 86.89 (4) and 88.01 (4)°.

### Experimental

The preparation is described by Schaller (1994). Suitable crystals were obtained by recrystallization from mixed hexanes.

### Refinement

All H atoms were placed in calculated positions guided by difference maps. The C—H bond distances were constrained to the range from 0.95 to 1.0 Å, and *U*_iso_= 1.2*U*_eq_ (1.5 for methyl groups), thereafter refined as riding. A torsional parameter was refined for each methyl group. One reflection was omitted from the refinement, because it was behind the beamstop and measured zero.

### Crystal data

**Table:**

C_32_H_28_	*Z* = 2
*M**_r_* = 412.54	*F*(000) = 440
Triclinic, *P*1	*D*_x_ = 1.235 Mg m^−^^3^
Hall symbol: -P 1	Mo *K*α radiation, λ = 0.71073 Å
*a* = 9.3577 (2) Å	Cell parameters from 7268 reflections
*b* = 9.5500 (3) Å	θ = 2.6–32.6°
*c* = 12.6946 (3) Å	µ = 0.07 mm^−^^1^
α = 94.068 (2)°	*T* = 90 K
β = 94.402 (2)°	Prism, colourless
γ = 100.162 (1)°	0.40 × 0.30 × 0.27 mm
*V* = 1109.24 (5) Å^3^	

### Data collection

**Table:**

Nonius KappaCCD diffractometer	7973 independent reflections
Radiation source: sealed tube	5906 reflections with *I* > 2σ(*I*)
Horizontally mounted graphite crystal monochromator	*R*_int_ = 0.033
Detector resolution: 9 pixels mm^-1^	θ_max_ = 32.6°, θ_min_ = 2.6°
CCD rotation images, thick slices scans	*h* = −14→13
Absorption correction: multi-scan (*SCALEPACK*; Otwinowski & Minor, 1997)	*k* = −14→14
*T*_min_ = 0.973, *T*_max_ = 0.982	*l* = −19→18
15041 measured reflections	

### Refinement

**Table:**

Refinement on *F*^2^	Primary atom site location: structure-invariant direct methods
Least-squares matrix: full	Secondary atom site location: difference Fourier map
*R*[*F*^2^ > 2σ(*F*^2^)] = 0.052	Hydrogen site location: inferred from neighbouring sites
*wR*(*F*^2^) = 0.140	H-atom parameters constrained
*S* = 1.04	*w* = 1/[σ^2^(*F*_o_^2^) + (0.0681*P*)^2^ + 0.3029*P*] where *P* = (*F*_o_^2^ + 2*F*_c_^2^)/3
7972 reflections	(Δ/σ)_max_ < 0.001
293 parameters	Δρ_max_ = 0.40 e Å^−^^3^
0 restraints	Δρ_min_ = −0.31 e Å^−^^3^

### Special details

**Table:**

Geometry. All e.s.d.'s (except the e.s.d. in the dihedral angle between two l.s. planes) are estimated using the full covariance matrix. The cell e.s.d.'s are taken into account individually in the estimation of e.s.d.'s in distances, angles and torsion angles; correlations between e.s.d.'s in cell parameters are only used when they are defined by crystal symmetry. An approximate (isotropic) treatment of cell e.s.d.'s is used for estimating e.s.d.'s involving l.s. planes.

### Fractional atomic coordinates and isotropic or equivalent isotropic displacement parameters (Å^2^)

**Table:**

	*x*	*y*	*z*	*U*_iso_*/*U*_eq_	
C1	0.51176 (11)	0.54105 (11)	0.17992 (8)	0.01164 (19)	
C2	0.51490 (12)	0.40789 (12)	0.12654 (8)	0.0145 (2)	
H2	0.4372	0.3305	0.1315	0.017*	
C3	0.62865 (12)	0.38631 (12)	0.06666 (9)	0.0162 (2)	
H3	0.6276	0.2956	0.0303	0.019*	
C4	0.74406 (12)	0.49844 (13)	0.06037 (9)	0.0163 (2)	
H4	0.8214	0.4856	0.0185	0.020*	
C5	0.74507 (12)	0.62937 (12)	0.11594 (8)	0.0142 (2)	
H5	0.8257	0.7046	0.1134	0.017*	
C6	0.63010 (11)	0.65370 (11)	0.17583 (8)	0.01175 (19)	
C7	0.64466 (11)	0.79423 (11)	0.23501 (8)	0.01148 (18)	
C8	0.55457 (11)	0.88949 (11)	0.23640 (8)	0.01190 (19)	
C9	0.41322 (11)	0.88530 (11)	0.17555 (8)	0.01217 (19)	
C10	0.39865 (12)	1.00158 (12)	0.11612 (9)	0.0159 (2)	
H10	0.4783	1.0791	0.1185	0.019*	
C11	0.27112 (13)	1.00619 (13)	0.05405 (9)	0.0184 (2)	
H11	0.2650	1.0843	0.0126	0.022*	
C12	0.15202 (13)	0.89528 (13)	0.05307 (9)	0.0180 (2)	
H12	0.0643	0.8969	0.0106	0.022*	
C13	0.16272 (12)	0.78247 (12)	0.11457 (9)	0.0149 (2)	
H13	0.0798	0.7093	0.1160	0.018*	
C14	0.29252 (11)	0.77331 (11)	0.17483 (8)	0.01192 (19)	
C15	0.29265 (11)	0.64752 (11)	0.23522 (8)	0.01116 (18)	
C16	0.38385 (11)	0.55264 (11)	0.23830 (8)	0.01137 (18)	
C17	0.31002 (12)	0.42739 (12)	0.29816 (8)	0.0140 (2)	
H17	0.3744	0.3641	0.3282	0.017*	
C18	0.23755 (12)	0.51417 (12)	0.37755 (8)	0.0147 (2)	
C19	0.15754 (11)	0.58407 (12)	0.29174 (8)	0.0140 (2)	
H19	0.0945	0.6519	0.3163	0.017*	
C20	0.08048 (12)	0.44649 (13)	0.22486 (9)	0.0162 (2)	
H20	−0.0139	0.4310	0.1878	0.019*	
C21	0.17094 (12)	0.35344 (12)	0.22873 (9)	0.0164 (2)	
H21	0.1532	0.2592	0.1951	0.020*	
C22	0.64166 (12)	1.02883 (12)	0.29767 (8)	0.0143 (2)	
H22	0.5837	1.0990	0.3263	0.017*	
C23	0.73077 (12)	0.95972 (12)	0.37897 (8)	0.0148 (2)	
C24	0.79219 (11)	0.87001 (12)	0.29476 (8)	0.0139 (2)	
H24	0.8601	0.8074	0.3208	0.017*	
C25	0.85602 (12)	0.99244 (12)	0.22954 (8)	0.0157 (2)	
H25	0.9429	0.9993	0.1946	0.019*	
C26	0.76665 (12)	1.08699 (12)	0.23115 (9)	0.0160 (2)	
H26	0.7779	1.1737	0.1977	0.019*	
C27	0.24121 (13)	0.52483 (13)	0.48278 (9)	0.0193 (2)	
C28	0.15625 (15)	0.61803 (15)	0.54365 (10)	0.0272 (3)	
H28A	0.0982	0.6642	0.4938	0.041*	
H28B	0.2239	0.6912	0.5897	0.041*	
H28C	0.0914	0.5594	0.5870	0.041*	
C29	0.33042 (15)	0.44386 (16)	0.55141 (10)	0.0276 (3)	
H29A	0.3817	0.3853	0.5065	0.041*	
H29B	0.2660	0.3821	0.5932	0.041*	
H29C	0.4016	0.5115	0.5993	0.041*	
C30	0.75090 (13)	0.97426 (13)	0.48432 (9)	0.0194 (2)	
C31	0.67789 (15)	1.07174 (15)	0.55135 (10)	0.0275 (3)	
H31A	0.6169	1.1202	0.5054	0.041*	
H31B	0.7522	1.1430	0.5938	0.041*	
H31C	0.6170	1.0157	0.5985	0.041*	
C32	0.85020 (15)	0.89543 (15)	0.54657 (10)	0.0267 (3)	
H32A	0.8932	0.8342	0.4976	0.040*	
H32B	0.7940	0.8365	0.5947	0.040*	
H32C	0.9279	0.9644	0.5878	0.040*	

### Atomic displacement parameters (Å^2^)

**Table:**

	*U*^11^	*U*^22^	*U*^33^	*U*^12^	*U*^13^	*U*^23^
C1	0.0123 (4)	0.0130 (5)	0.0102 (4)	0.0040 (3)	0.0001 (3)	0.0014 (3)
C2	0.0161 (5)	0.0129 (5)	0.0145 (5)	0.0032 (4)	0.0001 (4)	0.0005 (4)
C3	0.0195 (5)	0.0160 (5)	0.0140 (5)	0.0072 (4)	0.0006 (4)	−0.0006 (4)
C4	0.0154 (5)	0.0206 (5)	0.0141 (5)	0.0070 (4)	0.0024 (4)	−0.0002 (4)
C5	0.0123 (5)	0.0165 (5)	0.0142 (5)	0.0035 (4)	0.0015 (4)	0.0008 (4)
C6	0.0121 (4)	0.0131 (5)	0.0104 (4)	0.0037 (3)	0.0000 (3)	0.0008 (3)
C7	0.0121 (4)	0.0121 (5)	0.0100 (4)	0.0017 (3)	0.0010 (3)	0.0004 (3)
C8	0.0131 (4)	0.0115 (4)	0.0107 (4)	0.0009 (3)	0.0017 (3)	0.0006 (3)
C9	0.0141 (5)	0.0121 (5)	0.0110 (4)	0.0042 (4)	0.0020 (3)	−0.0002 (3)
C10	0.0191 (5)	0.0133 (5)	0.0159 (5)	0.0039 (4)	0.0028 (4)	0.0024 (4)
C11	0.0232 (6)	0.0165 (5)	0.0179 (5)	0.0085 (4)	0.0022 (4)	0.0046 (4)
C12	0.0174 (5)	0.0221 (6)	0.0163 (5)	0.0098 (4)	−0.0008 (4)	0.0023 (4)
C13	0.0133 (5)	0.0172 (5)	0.0149 (5)	0.0050 (4)	0.0014 (4)	0.0003 (4)
C14	0.0132 (4)	0.0130 (5)	0.0104 (4)	0.0045 (3)	0.0027 (3)	0.0000 (3)
C15	0.0107 (4)	0.0129 (5)	0.0097 (4)	0.0018 (3)	0.0008 (3)	0.0003 (3)
C16	0.0117 (4)	0.0116 (5)	0.0102 (4)	0.0009 (3)	−0.0002 (3)	0.0006 (3)
C17	0.0146 (5)	0.0136 (5)	0.0133 (5)	0.0009 (4)	0.0008 (4)	0.0033 (4)
C18	0.0127 (5)	0.0165 (5)	0.0137 (5)	−0.0011 (4)	0.0013 (4)	0.0024 (4)
C19	0.0125 (5)	0.0168 (5)	0.0126 (4)	0.0018 (4)	0.0023 (4)	0.0016 (4)
C20	0.0133 (5)	0.0200 (5)	0.0135 (5)	−0.0010 (4)	−0.0002 (4)	0.0019 (4)
C21	0.0173 (5)	0.0150 (5)	0.0150 (5)	−0.0015 (4)	0.0004 (4)	0.0007 (4)
C22	0.0164 (5)	0.0126 (5)	0.0133 (5)	0.0008 (4)	0.0022 (4)	−0.0005 (4)
C23	0.0142 (5)	0.0151 (5)	0.0132 (5)	−0.0017 (4)	0.0018 (4)	−0.0002 (4)
C24	0.0127 (5)	0.0152 (5)	0.0129 (4)	0.0009 (4)	0.0003 (4)	0.0012 (4)
C25	0.0145 (5)	0.0191 (5)	0.0122 (5)	−0.0009 (4)	0.0023 (4)	0.0007 (4)
C26	0.0182 (5)	0.0147 (5)	0.0135 (5)	−0.0019 (4)	0.0022 (4)	0.0010 (4)
C27	0.0185 (5)	0.0215 (6)	0.0145 (5)	−0.0058 (4)	0.0018 (4)	0.0025 (4)
C28	0.0304 (7)	0.0296 (7)	0.0171 (5)	−0.0074 (5)	0.0101 (5)	−0.0049 (5)
C29	0.0270 (6)	0.0345 (7)	0.0164 (5)	−0.0080 (5)	−0.0046 (5)	0.0105 (5)
C30	0.0197 (5)	0.0214 (6)	0.0130 (5)	−0.0072 (4)	0.0016 (4)	−0.0001 (4)
C31	0.0300 (7)	0.0310 (7)	0.0159 (5)	−0.0082 (5)	0.0078 (5)	−0.0080 (5)
C32	0.0286 (7)	0.0311 (7)	0.0150 (5)	−0.0076 (5)	−0.0059 (5)	0.0057 (5)

### Geometric parameters (Å, º)

**Table:**

C1—C2	1.4040 (15)	C18—C27	1.3298 (15)
C1—C6	1.4081 (14)	C18—C19	1.5320 (15)
C1—C16	1.4716 (14)	C19—C20	1.5429 (16)
C2—C3	1.3902 (15)	C19—H19	1.0000
C2—H2	0.9500	C20—C21	1.3319 (17)
C3—C4	1.3917 (16)	C20—H20	0.9500
C3—H3	0.9500	C21—H21	0.9500
C4—C5	1.3896 (16)	C22—C23	1.5346 (16)
C4—H4	0.9500	C22—C26	1.5422 (15)
C5—C6	1.4064 (14)	C22—H22	1.0000
C5—H5	0.9500	C23—C30	1.3301 (15)
C6—C7	1.4701 (15)	C23—C24	1.5314 (15)
C7—C8	1.3451 (15)	C24—C25	1.5414 (15)
C7—C24	1.5536 (15)	C24—H24	1.0000
C8—C9	1.4724 (14)	C25—C26	1.3348 (17)
C8—C22	1.5533 (15)	C25—H25	0.9500
C9—C10	1.4062 (15)	C26—H26	0.9500
C9—C14	1.4108 (15)	C27—C29	1.5052 (18)
C10—C11	1.3879 (16)	C27—C28	1.5060 (18)
C10—H10	0.9500	C28—H28A	0.9800
C11—C12	1.3941 (17)	C28—H28B	0.9800
C11—H11	0.9500	C28—H28C	0.9800
C12—C13	1.3877 (16)	C29—H29A	0.9800
C12—H12	0.9500	C29—H29B	0.9800
C13—C14	1.4045 (15)	C29—H29C	0.9800
C13—H13	0.9500	C30—C31	1.5029 (18)
C14—C15	1.4702 (15)	C30—C32	1.5077 (19)
C15—C16	1.3505 (14)	C31—H31A	0.9800
C15—C19	1.5528 (14)	C31—H31B	0.9800
C16—C17	1.5503 (15)	C31—H31C	0.9800
C17—C18	1.5321 (15)	C32—H32A	0.9800
C17—C21	1.5430 (15)	C32—H32B	0.9800
C17—H17	1.0000	C32—H32C	0.9800
			
C2—C1—C6	118.81 (9)	C18—C19—H19	116.9
C2—C1—C16	117.03 (9)	C20—C19—H19	116.9
C6—C1—C16	124.16 (9)	C15—C19—H19	116.9
C3—C2—C1	121.75 (10)	C21—C20—C19	107.19 (9)
C3—C2—H2	119.1	C21—C20—H20	126.4
C1—C2—H2	119.1	C19—C20—H20	126.4
C2—C3—C4	119.54 (10)	C20—C21—C17	107.18 (10)
C2—C3—H3	120.2	C20—C21—H21	126.4
C4—C3—H3	120.2	C17—C21—H21	126.4
C5—C4—C3	119.35 (10)	C23—C22—C26	97.95 (9)
C5—C4—H4	120.3	C23—C22—C8	97.91 (8)
C3—C4—H4	120.3	C26—C22—C8	107.32 (8)
C4—C5—C6	121.88 (10)	C23—C22—H22	116.9
C4—C5—H5	119.1	C26—C22—H22	116.9
C6—C5—H5	119.1	C8—C22—H22	116.9
C5—C6—C1	118.61 (10)	C30—C23—C24	132.56 (11)
C5—C6—C7	117.50 (9)	C30—C23—C22	133.28 (11)
C1—C6—C7	123.81 (9)	C24—C23—C22	94.14 (8)
C8—C7—C6	130.92 (10)	C23—C24—C25	97.84 (9)
C8—C7—C24	106.93 (9)	C23—C24—C7	97.76 (8)
C6—C7—C24	121.31 (9)	C25—C24—C7	107.36 (8)
C7—C8—C9	130.01 (10)	C23—C24—H24	116.9
C7—C8—C22	106.74 (9)	C25—C24—H24	116.9
C9—C8—C22	122.04 (9)	C7—C24—H24	116.9
C10—C9—C14	118.72 (10)	C26—C25—C24	107.36 (9)
C10—C9—C8	117.34 (10)	C26—C25—H25	126.3
C14—C9—C8	123.94 (9)	C24—C25—H25	126.3
C11—C10—C9	121.78 (11)	C25—C26—C22	106.97 (10)
C11—C10—H10	119.1	C25—C26—H26	126.5
C9—C10—H10	119.1	C22—C26—H26	126.5
C10—C11—C12	119.44 (11)	C18—C27—C29	122.74 (12)
C10—C11—H11	120.3	C18—C27—C28	123.12 (12)
C12—C11—H11	120.3	C29—C27—C28	114.14 (11)
C13—C12—C11	119.46 (10)	C27—C28—H28A	109.5
C13—C12—H12	120.3	C27—C28—H28B	109.5
C11—C12—H12	120.3	H28A—C28—H28B	109.5
C12—C13—C14	121.90 (10)	C27—C28—H28C	109.5
C12—C13—H13	119.0	H28A—C28—H28C	109.5
C14—C13—H13	119.0	H28B—C28—H28C	109.5
C13—C14—C9	118.60 (10)	C27—C29—H29A	109.5
C13—C14—C15	117.35 (10)	C27—C29—H29B	109.5
C9—C14—C15	124.04 (9)	H29A—C29—H29B	109.5
C16—C15—C14	131.01 (9)	C27—C29—H29C	109.5
C16—C15—C19	106.86 (9)	H29A—C29—H29C	109.5
C14—C15—C19	121.04 (9)	H29B—C29—H29C	109.5
C15—C16—C1	130.80 (9)	C23—C30—C31	122.88 (12)
C15—C16—C17	106.60 (9)	C23—C30—C32	122.80 (12)
C1—C16—C17	121.28 (9)	C31—C30—C32	114.31 (11)
C18—C17—C21	97.97 (8)	C30—C31—H31A	109.5
C18—C17—C16	98.11 (8)	C30—C31—H31B	109.5
C21—C17—C16	107.16 (8)	H31A—C31—H31B	109.5
C18—C17—H17	116.8	C30—C31—H31C	109.5
C21—C17—H17	116.8	H31A—C31—H31C	109.5
C16—C17—H17	116.8	H31B—C31—H31C	109.5
C27—C18—C19	132.61 (11)	C30—C32—H32A	109.5
C27—C18—C17	133.24 (11)	C30—C32—H32B	109.5
C19—C18—C17	94.15 (8)	H32A—C32—H32B	109.5
C18—C19—C20	97.99 (9)	C30—C32—H32C	109.5
C18—C19—C15	97.64 (8)	H32A—C32—H32C	109.5
C20—C19—C15	107.39 (9)	H32B—C32—H32C	109.5
			
C6—C1—C2—C3	−2.57 (15)	C15—C16—C17—C21	66.01 (11)
C16—C1—C2—C3	178.23 (10)	C1—C16—C17—C21	−102.21 (11)
C1—C2—C3—C4	1.03 (16)	C21—C17—C18—C27	125.62 (13)
C2—C3—C4—C5	1.34 (16)	C16—C17—C18—C27	−125.66 (13)
C3—C4—C5—C6	−2.18 (16)	C21—C17—C18—C19	−53.94 (9)
C4—C5—C6—C1	0.63 (15)	C16—C17—C18—C19	54.78 (9)
C4—C5—C6—C7	177.63 (10)	C27—C18—C19—C20	−125.66 (13)
C2—C1—C6—C5	1.71 (15)	C17—C18—C19—C20	53.90 (9)
C16—C1—C6—C5	−179.16 (9)	C27—C18—C19—C15	125.47 (13)
C2—C1—C6—C7	−175.09 (9)	C17—C18—C19—C15	−54.97 (9)
C16—C1—C6—C7	4.04 (16)	C16—C15—C19—C18	36.15 (10)
C5—C6—C7—C8	127.28 (12)	C14—C15—C19—C18	−154.55 (9)
C1—C6—C7—C8	−55.89 (16)	C16—C15—C19—C20	−64.75 (11)
C5—C6—C7—C24	−40.80 (13)	C14—C15—C19—C20	104.55 (11)
C1—C6—C7—C24	136.03 (10)	C18—C19—C20—C21	−35.12 (11)
C6—C7—C8—C9	−2.37 (19)	C15—C19—C20—C21	65.51 (11)
C24—C7—C8—C9	166.99 (10)	C19—C20—C21—C17	−0.07 (12)
C6—C7—C8—C22	−169.67 (10)	C18—C17—C21—C20	35.23 (11)
C24—C7—C8—C22	−0.31 (11)	C16—C17—C21—C20	−65.85 (11)
C7—C8—C9—C10	−125.05 (12)	C7—C8—C22—C23	−35.24 (10)
C22—C8—C9—C10	40.57 (14)	C9—C8—C22—C23	156.22 (9)
C7—C8—C9—C14	55.85 (16)	C7—C8—C22—C26	65.68 (11)
C22—C8—C9—C14	−138.53 (10)	C9—C8—C22—C26	−102.86 (11)
C14—C9—C10—C11	−2.48 (16)	C26—C22—C23—C30	124.46 (13)
C8—C9—C10—C11	178.38 (10)	C8—C22—C23—C30	−126.71 (13)
C9—C10—C11—C12	2.28 (17)	C26—C22—C23—C24	−54.13 (9)
C10—C11—C12—C13	0.31 (17)	C8—C22—C23—C24	54.71 (9)
C11—C12—C13—C14	−2.71 (17)	C30—C23—C24—C25	−124.57 (13)
C12—C13—C14—C9	2.48 (16)	C22—C23—C24—C25	54.04 (9)
C12—C13—C14—C15	−178.66 (10)	C30—C23—C24—C7	126.61 (13)
C10—C9—C14—C13	0.11 (15)	C22—C23—C24—C7	−54.79 (9)
C8—C9—C14—C13	179.19 (9)	C8—C7—C24—C23	35.81 (10)
C10—C9—C14—C15	−178.68 (9)	C6—C7—C24—C23	−153.58 (9)
C8—C9—C14—C15	0.41 (16)	C8—C7—C24—C25	−64.96 (11)
C13—C14—C15—C16	127.68 (12)	C6—C7—C24—C25	105.65 (11)
C9—C14—C15—C16	−53.52 (16)	C23—C24—C25—C26	−35.32 (11)
C13—C14—C15—C19	−38.70 (14)	C7—C24—C25—C26	65.40 (11)
C9—C14—C15—C19	140.10 (10)	C24—C25—C26—C22	0.02 (12)
C14—C15—C16—C1	−1.89 (19)	C23—C22—C26—C25	35.21 (11)
C19—C15—C16—C1	165.94 (10)	C8—C22—C26—C25	−65.69 (11)
C14—C15—C16—C17	−168.56 (10)	C19—C18—C27—C29	−179.81 (11)
C19—C15—C16—C17	−0.73 (11)	C17—C18—C27—C29	0.8 (2)
C2—C1—C16—C15	−128.07 (12)	C19—C18—C27—C28	0.5 (2)
C6—C1—C16—C15	52.79 (16)	C17—C18—C27—C28	−178.90 (11)
C2—C1—C16—C17	36.95 (13)	C24—C23—C30—C31	179.52 (11)
C6—C1—C16—C17	−142.20 (10)	C22—C23—C30—C31	1.4 (2)
C15—C16—C17—C18	−34.98 (10)	C24—C23—C30—C32	0.2 (2)
C1—C16—C17—C18	156.80 (9)	C22—C23—C30—C32	−177.91 (11)
